# Circuit-specific and neuronal subcellular-wide E-I balance in cortical pyramidal cells

**DOI:** 10.1038/s41598-018-22314-9

**Published:** 2018-03-05

**Authors:** Weiguo Yang, Qian-Quan Sun

**Affiliations:** Department of Zoology and Physiology, University of Wyoming, Laramie, WY 82071 USA

## Abstract

We used ChR2-assisted circuit mapping (CRACM) to examine neuronal/compartmental excitatory and inhibitory synaptic balance (E-I balance) in pyramidal cells (PCs) located in several brain regions (including both neocortices and paleocortices). Within the vS1, different inputs on the same neurons, or the same inputs formed on different targets, induced different E/I ratios. E/I ratios in PCs from different regions were largely different. Chemogenetic silencing of somatostatin (SOM)- or parvalbumin (PV)-containing interneurons (INs) while optogenetically activating long-range M1 inputs demonstrated differential contribution of PV and SOM INs to the E/I ratios in a layer-specific manner in S1. Our results thus demonstrate that there are both universal subcellular-wide E-I balance within single PC and high specificity in the value of E/I ratios across different circuits (i.e. visual, somatosensory, piriform and hippocampal). Specificity of E/I balance are likely caused by unique glutamatergic innervation of interneurons. The dichotomy of high specificity and generalization of subcellular E-I balance in different circuits forms the basis for further understanding of neuronal computation under physiological conditions and various neuro-psychiatric disease-states.

## Introduction

Excitatory and inhibitory synaptic transmissions in cortical circuits form the basis of neural communication and computation^1^. Stimulation of a single glutamatergic pyramidal cell (PC) in cerebral cortex activates selected downstream cells through mono- and poly-synaptic connections^2–4^, leading to appropriate behavioral output^5^. The phenomenon of excitation and inhibition synaptic balance (E-I balance), defined by matched glutamatergic and GABAergic synaptic strength following neural activation, has been widely reported in many different brain regions,cell types and animal models. Theoretical and experimental studies show that the neocortical network activity *in vivo* is generated through a dynamic E-I balance^6–10^. The E/I ratio of PCs induced by activation of synaptic inputs in sensory cortices is regulated to avoid runaway excitation or quiescence in response to variable inputs^11–13^. The co-occurrence of synaptic excitation and inhibition is critical to sensory perception and neuronal output^14–16^, help increase temporal precision and reduce the randomness of cortical operation^11,13,17,18^. Balance between recurrent excitation and feedforward (or feedback) inhibition can also optimize network amplification^19,20^ or stability^21^. The relative balanced synaptic strength at different subcellular compartments, manifested as a specific E/I ratio, may underlie the logic of circuit organization and computation at subcellular levels (e.g. soma vs. dendrites).

Despite the wide recognition of the E-I balance and available data from various brain regions, horizontal comparisons between different inputs to a particular brain region, vertical comparisons between different layers receiving same inputs, and macroscopic (i.e. brain-region wide) and microscopic (i.e. subcellular wide) scale comparisons of the E-I balance have not been widely reported. Such comparisons may help further understand the specificity and/or generalization of E-I balance and reveal some fundamental principles governing the establishment of E-I balance at single PC. The understanding of E-I balance is of critically importance, because unbalanced E-I has been attributed to as a critical cellular basis underlying major neurological and psychiatric disorders^22^, such as epilepsy^23–25^, autism^26^, schizophrenia and other neuropsychiatric disorders^27^. However, it is unclear whether a disturbance of specific features of E-I balance is associated with particular cognitive or behavior phenotypes of the neurological and neuropsychiatric diseases. In order to further advance this field, it is of paramount importance to understand the key features of E-I balance in wide range of cortical circuits in normal brains first.

Recent technological advances make it possible to record electrophysiological responses from a genetically identified cell type upon activation of a precisely defined presynaptic input^28–32^. Furthermore, the CRACM method enables us to study the spatial pattern of synaptic inputs at cellular/subcellular level^33–35^. Our goal here is to determine whether the E/I ratio of a cortical PC differ for different presynaptic inputs and whether there is any common feature of E-I balance across multiple selected brain regions. Our results demonstrate that there are both cell-wide E-I balance across different subcellular compartments in all brain regions (i.e. generalization) and high specificity in the value of E/I ratios that presumably arise from unique synaptic innervation patterns in each region. These novel results imply a fundamentally important but untested hypothesis: i.e. there are coordinated excitatory and inhibitory synaptic wiring across various cortical layers/columns and modalities, which may underlie circuit-specific neural computations.

## Results

### Subcellular E-I balance associated with activation of canonical sensory feedforward circuits in vS1

**Figure 1:**
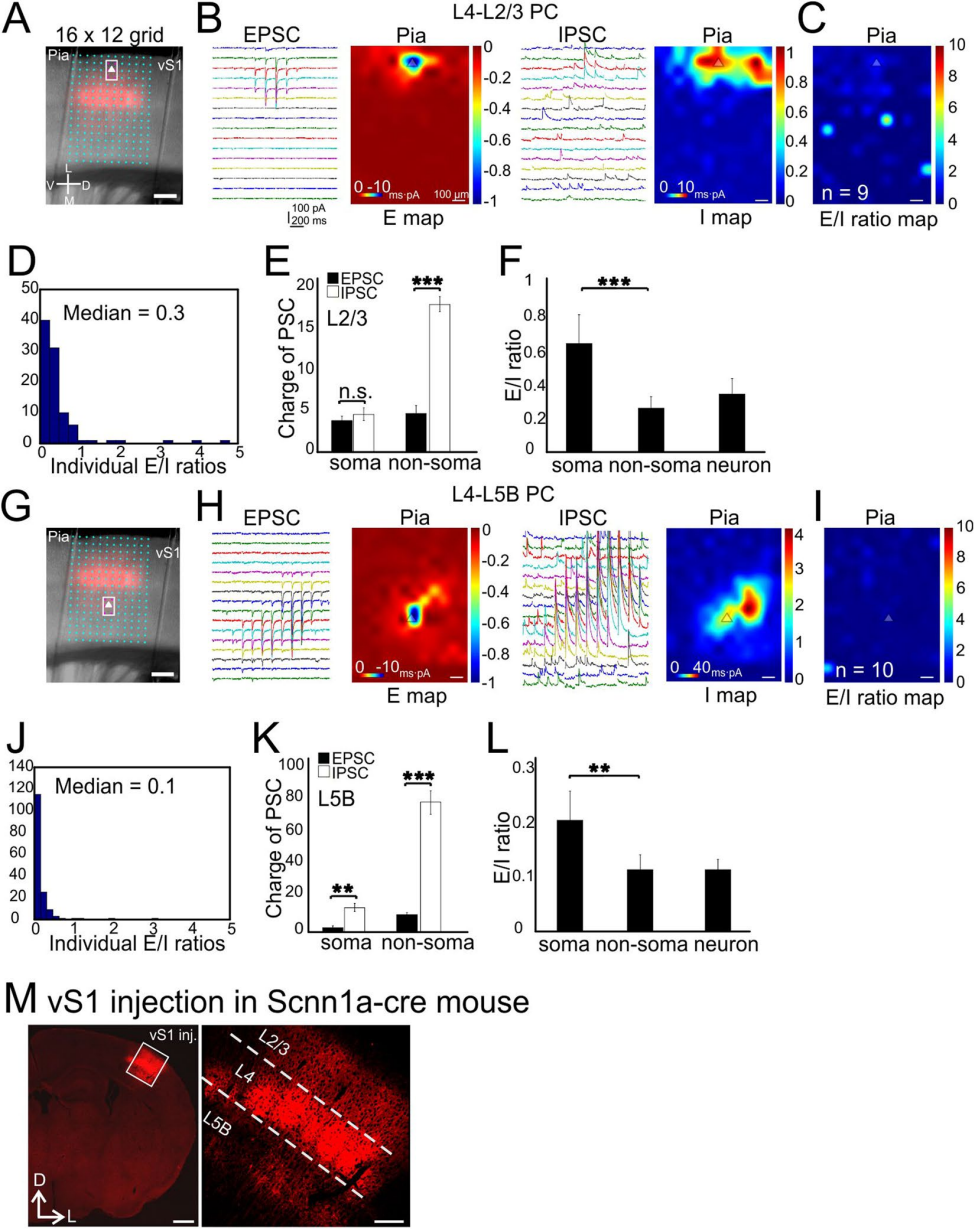
CRACM of L4-specific synapses on L2/3 and L5B PCs in vS1. (**A**) A 16 × 12 photostimulation grid (75 µm spacing) overlaid on a coronal slice. Pink color indicates barrels expressing mCherry fluorescent protein. The triangle indicates the soma position of an example of recorded L2/3 PC. The boxed six pixels surrounding the soma were defined as the “soma” region. Scale bar, 200 µm. (**B**) Traces: examples of EPSC/IPSC recorded from a single L2/3 PC. Heat maps: averaged CRACM input maps aligned by soma position (triangle). Excitatory (**E**) maps and inhibitory (**I**) maps were recorded at reversal potentials for inhibitory synaptic currents and excitatory synaptic currents, respectively. The inset scales represent the actual recorded PSC charge values and the outside scales for the E and I maps were normalized to the maximum E charge within maps. *n* = 9 cells, 5 mice. Scale bar: 100 µm for all the heat maps hereafter. (**C**) Group averages of the E/I ratio maps across pixels. Scale bar, 300 µm, applying to all heat maps hereafter. (**D**) Quantitative distribution of pixel E/I ratios shown in the panel C. (**E**) Comparisons of absolute charges between EPSCs and IPSCs for both soma and non-soma regions. (**F**) E/I ratios at compartmental soma, non-soma regions and of the whole neurons. (**G**–**L**) CRACM of L4-L5B PCs. Legend layout similar as above. *n* = 10 cells, 5 mice. *P < 0.001, n.s., not statistically significant. (**M**) Flex-virus injection in vS1 with Scnn1a-cre mouse. Left, low-magnification image of ChR2-mChe expressing in vS1 glutamatergic neurons; right, zoom in image. Scale bar left: 0.5 mm, scale bar right: 200 µm.

We first investigated the E-I balance associated with activation of inter-laminar glutamatergic synaptic inputs to PCs in whisker-related primary somatosensory cortex (vS1). This was achieved via using a genetically-defined presynaptic source: vS1 layer 4 (L4). PCs in L4 were transfected with AAV-flex-ChR2 in Scnn1a-Tg3-cre mice (Fig. 1A,M). Individually labeled L4 spiny stellate cells project predominantly to L2/3 and secondarily to L5B. The combination of viral vector and Cre recombinase allows us to examine E-I balance associated with optogenetic activation of L4→L2/3 and L4→L5B connections inputs, respectively. This synaptic pathway is normally activated during whisker-related sensory processing upon thalamocortical activation of L4 neurons. A 16 by 12 photo-stimulation grid (with 75 µm spacing), essentially covering around 5 barrel columns (Fig. 1A, Materials and Methods), was used to map the blue laser-induced synaptic responses. We first examined E-I balance associated with optogenetic activation L4→L2/3 inputs in L2/3 PCs. E and I map were obtained by voltage-clamp of recorded cells under the reversal potential for GABA and glutamate, respectively (see Materials and Methods). The E and I maps were confirmed to be mediated by ionotropic glutamate and GABA_A_ receptors, respectively through pharmacologically blocking these receptors with NBQX and GABAzine applications, respectively (data not shown). E/I ratio = charge of EPSC/charge of IPSC. E/I was then calculated at each pixel location (total of 16 × 12 = 192 pixels per scanned image, e.g. Fig. 1C, see Material and Methods). The median value of individual E/I ratios in all 9 cells was 0.3 (Fig. 1D, *n* = 9 cells from 5 mice). To examine if there were compartmentalized E-I balance at different subcellular locations, we defined six pixels surrounding recorded cells as the “soma” area (indicated with the boxed magenta colored spots in Fig. 1A,G) and the rest as the “non-soma” region. Our results showed that the charge of EPSC and IPSC at individual pixels matched with each other at the soma region (Fig. 1E, 3.5 ± 0.7 and 4.4 ± 1.0 for EPSC and IPSC, respectively). At the non-soma region, the charge of the IPSCs were much larger than that of EPSC (Fig. 1E, 4.35 ± 0.61 and 17.7 ± 0.89 for EPSC and IPSC, non-parametric Wilcoxon signed-rank test, *P* < 0.001, same statistics hereafter). This large difference are likely resulted from strong di-synaptic glutamatergic recruitment of lateral inhibitory innervation of soma region from adjacent barrel columns (Fig. 1B, right). Thus the mean E/I ratios for soma and non-soma region were significantly different, with the relative values of 0.8 ± 0.2 and 0.3 ± 0.1, respectively (Fig. 1F, *P* < 0.001). The combined neuronal E/I ratio (total E charge divided by total I charge, Materials and Methods) was 0.3 ± 0.1 for the L4→L2/3 connections (Fig. 1F).

In contrast, E-I balance associated with the activation of the L4→L5B inputs in L5B PCs is biased more towards stronger inhibition (Fig. 1G,H, note the scale differences for E and I maps). Similar to L4 →L2/3 E-I balance, L4 → L5B E-I balance showed strong di-synaptic inhibition from adjacent columns. The E/I ratios at individual pixels in L5B PCs were significantly lower than that of L4→L2/3 connections in L2/3 PCs with a median value of 0.1 (Fig. 1I,J, *n* = 10 cells, 5 mice, *P* < 0.01). The IPSC charge significantly exceeded EPSC charge at both the soma (Fig. 1K, 2.5 ± 0.7 and 12.3 ± 5.3 for EPSC and IPSC, *P* < 0.01) and non-soma region (Fig. 1K, 7.1 ± 0.6 and 76.7 ± 4.9 for EPSC and IPSC, *P* < 0.001). The E/I ratios at the soma and non-soma region were 0.2 ± 0.0 and 0.1 ± 0.0 (Fig. 1L, *P* < 0.01), respectively, with a combined neuronal E/I ratio of 0.1. In summary, activation of L4 inputs in L2/3 PCs induced E/I ratios much higher than those in L5B PCs. L4-induced E/I ratios were significantly higher at the soma regions than the non-soma region in L2/3 but not L5B PCs. Thus, the same cohort of inputs (i.e. from vS1 L4) mediated targets (i.e. L2/3 vs. L5B PCs) and subcellular (somatic vs. non-somatic) specific E-I balances in the canonical sensory feedforward circuits of the barrel cortex.

### Subcellular E-I balance associated with activation of intracolumnar non-specific synaptic inputs in vS1

**Figure 2:**
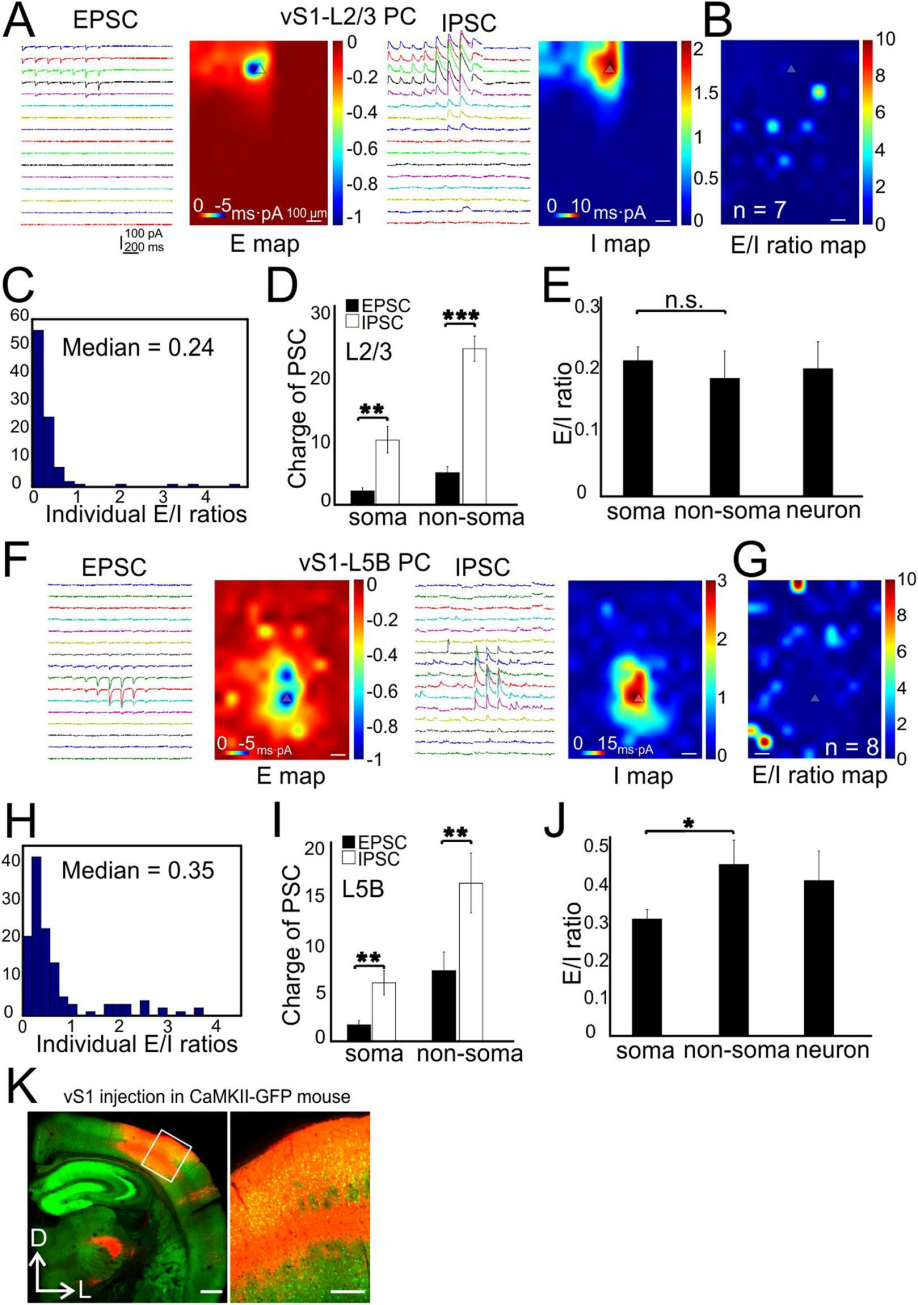
CRACM of vS1 local synapses on L2/3 and L5B PCs in vS1. (**A**) Traces: examples of EPSC/IPSC recorded from a single L2/3 PC. Heat maps: averaged CRACM input maps aligned by soma position. (**B**) Group averages of the E/I ratio maps across pixels. *n* = 7 cells, 4 mice. (**C**) Quantitative distribution of pixel E/I ratios shown in panel C. (**D**) Comparisons of absolute charges between EPSCs and IPSCs for both soma and dendrites regions. (**E**) E/I ratios at compartmental soma, dendrites regions and of the whole neurons. (**F**–**J**) CRACM of vS1-L5B PCs. Legend layout similar as above. *n* = 8 cells, 4 mice. *P < 0.05, **P < 0.01, ***P < 0.001. (**K**) Virus injection in vS1 with CaMKII-GFP mouse. Left, low-magnification image of ChR2-mChe expressing in vS1 neurons; right, zoom in image. Scale bar left: 0.5 mm, scale bar right: 200 µm.

Next, we expressed AAV-CAG-ChR2 in vS1 to transfect both excitatory and inhibitory neurons to study E-I balance associated with the activation of mixed local intracortical synaptic inputs to postsynaptic ChR2-negative L2/3 and L5B PCs in vS1 (Fig. 2K). Typically, 5 entire barrel columns (~1 mm width) were transfected with ChR2 (e.g. Fig. 2K). The CRACM in L2/3 PCs showed that the averaged E map roughly matched with the I map (Fig. 2B), generating an E/I ratio with a median value of 0.2 (Fig. 2C, *n* = 7 cells from 4 mice). The IPSC charges significantly exceeded EPSC charges at both the soma (Fig. 2D, 2.2 ± 0.6 and 10.3 ± 2.9 for EPSC and IPSC, *P* < 0.01) and the non-soma regions (Fig. 2D, 4.5 ± 0.5 and 22.4 ± 2.3 for EPSC and IPSC, *P* < 0.001) regions. The E/I ratios at soma and non-soma regions were similar: 0.2 ± 0.0 and 0.18 ± 0.0, respectively (Fig. 2E, *P* = 0.29). The mean neuronal E/I ratio was 0.2. The CRACM of L5B PCs indicated spatially more extensive excitatory inputs (Fig. 2F) compared to those of L2/3 PCs. The L5B E/I ratios at individual pixels were also higher compared to those of L2/3 PCs, with a median value of 0.35 *vs* 0.24 (Fig. 2H,I, *n* = 8 cells from 4 mice, *P* < 0.05). Similar to L2/3 PCs, the IPSC charge were larger than the EPSC charge at both the soma (Fig. 2I, 1.9 ± 0.6 and 6.1 ± 1.7 for EPSC and IPSC, *P* < 0.01) and non-soma regions (Fig. 2I, 6.9 ± 0.5 and 15.7 ± 1.6 for EPSC and IPSC, *P* < 0.01). The E/I ratios at non-soma regions were significantly higher than soma regions (Fig. 2J, 0.45 ± 0.1 and 0.3 ± 0.0 respectively, *P* < 0.05). The mean neuronal E/I ratio was 0.4. These results indicate that intracolumnar local circuits in vS1 may operate in a different way than the canonical sensory related feedforward L4→L2/3 and L4→L5B circuits: the net effect of activation of the vS1 intracolumnar local inputs tends to silence L2/3 PCs; in contrast, canonical sensory feedforward L4 inputs mainly activates L2/3 PCs.

### Subcellular E-I balance associated with activation long-range synaptic inputs in vS1 and visual cortex

**Figure 3:**
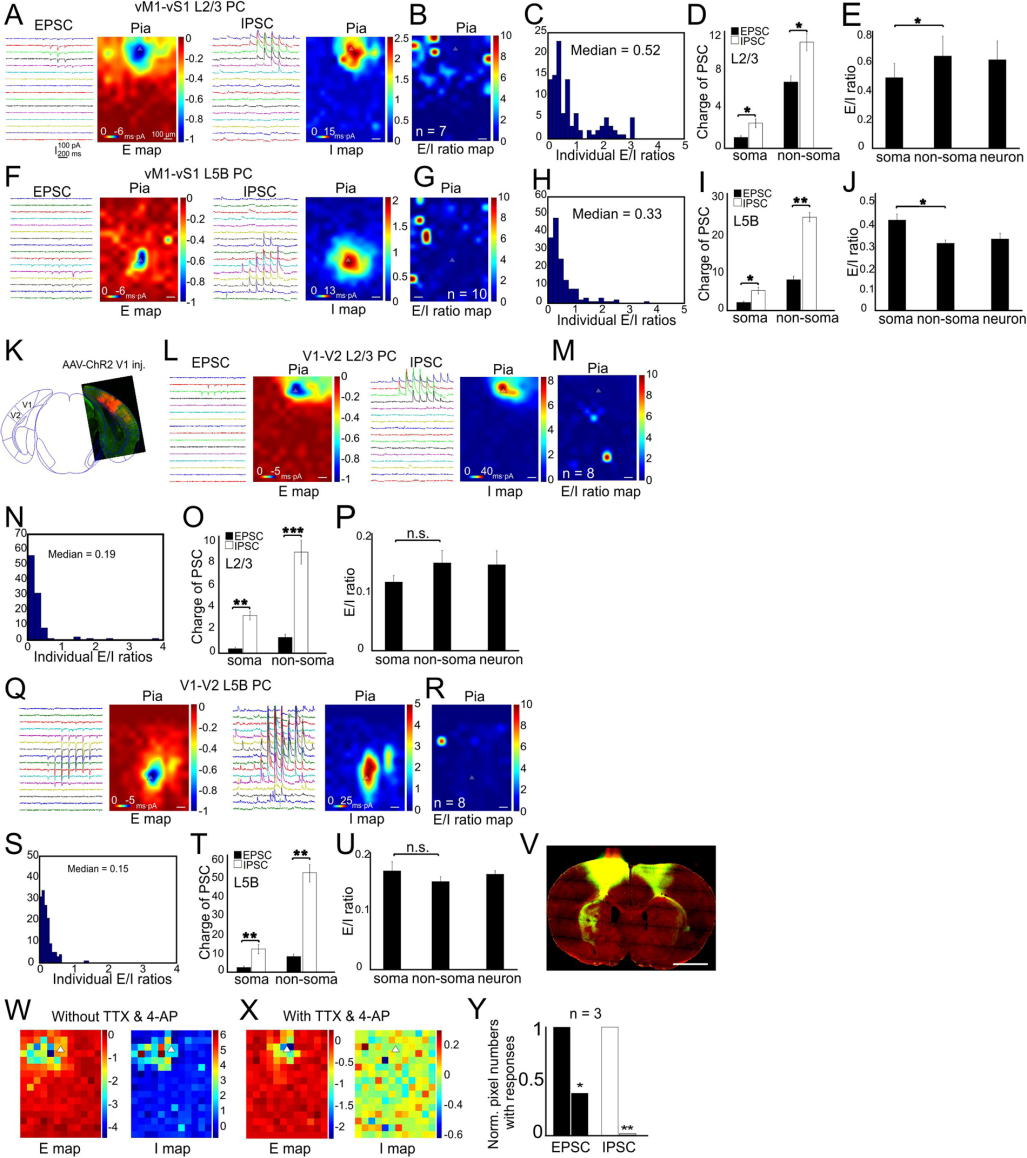
CRACM of long-range vM1 synapses on L2/3 and L5B PCs in vS1. (**A**–**E**) CRACM of vM1-vS1 L2/3 PCs in vS1. (**A**) Traces: examples of EPSC/IPSC recorded from a single L2/3 PC. Heat maps: averaged CRACM input maps aligned by soma position. (**B**) Group averages of the E/I ratio maps across pixels. *n* = 7 cells, 3 mice. (**C**) Quantitative distribution of pixel E/I ratios shown in panel C. (**D**) Comparisons of absolute charges between EPSCs and IPSCs for both soma and dendrites regions. (**E**) E/I ratios at compartmental soma, dendrites regions and of the whole neurons. (**F**–**I**) CRACM of vM1-vS1 L5B PCs. Legend layout similar as above. *n* = 10 cells, 3 mice. (**J**–**T**) V1 synapses on L2/3 and L5B PCs in V2. (**J**) Overlay of a coronal slice with an atlas schematic showing AAV-ChR2 injection in V1 and projection in V2 (Bregma: −3.64 mm, Paxinos and Franklin, 1997). (**K**–**O**) CRACM of V1-V2 L2/3 PCs. Legend layout similar as above. *n* = 8 cells, 3 mice. (P-T) CRACM of V1-V2 L5B PCs. Legend layout similar as above. *n* = 8 cells, 3 mice. *P < 0.05, **P < 0.01, ***P < 0.001. (**U**) Virus injection in vM1 with GAD67-GFP mouse. Left, low-magnification image of ChR2-mChe expressing in vM1 injected site; middle, vS1 projection; right, biotin-filled L5B PCs during CRACM experiments. (**V**–**X**) Examples of E and I maps with the presence of TTX and 4AP. Note the di-synaptic inhibition was gone in the panel W. Scale bar in V: 1 mm.

To examine subcellular E-I balances associated with long-range synaptic inputs, we first expressed AAV-CAG-ChR2 in primary motor cortex (vM1) regions and mapped the vM1→vS1 long-range glutamatergic connections on both postsynaptic L2/3 and L5B PCs in vS1 (Fig. 3V). The CRACM of vS1 L2/3 PCs showed extensive excitatory and inhibitory responses resulting from activation vM1 axon terminals in vS1 (Fig. 3A). The median E/I ratio across individual pixels was 0.5 (Fig. 3B,C, *n* = 7 cells from 3 mice). Inhibition was significantly stronger than the corresponding excitation at both soma (Fig. 3D, 1.2 ± 0.1 and 2.4 ± 1.7 for EPSC and IPSC, *P* < 0.05) and non-soma regions (Fig. 3E, 6.8 ± 0.3 and 10.9 ± 0.8 for EPSC and IPSC, *P* < 0.05). The E/I ratio at non-soma regions was significantly higher than that of the soma regions (Fig. 3E, 0.6 ± 0.2 *vs* 0.5 ± 0.1, *P* < 0.05). The overall neuronal E/I ratio was 0.6. The CRACM of vS1 L5B PCs indicated spatially limited excitatory inputs (Fig. 3F left vs. 3 A left). L5B PCs had a lower median E/I ratio of 0.3 compared to L2/3 PCs (0.5, Fig. 3H, *n* = 10 cells from 3 mice, *P* < 0.05). The IPSC charges were significantly higher than the EPSC charges at both the soma (Fig. 3I, 1.8 ± 0.3 and 4.4 ± 0.9 for EPSC and IPSC, P < 0.05) and the non-soma regions (Fig. 3I, 7.5 ± 0.5 and 24.1 ± 1.7 for EPSC and IPSC, *P* < 0.01). In contrast to L2/3 PCs, the E/I ratio at L5B PC soma region was higher than that at non-soma regions (Fig. 3J, 0.4 ± 0.0 *vs* 0.3 ± 0.0, *P* < 0.05), suggesting target-specific differences in the compartmentalized E/I ratios associated with activation of long-range vM1→vS1 synapses.

To test whether differential distribution of E/I ratios are associated with other long-range connections in secondary sensory cortices, we next expressed AAV-CAG-ChR2 in primary visual cortex (V1, Fig. 3K) and mapped the V1→V2 (higher lateromedial extrastriate area) synapses on both L2/3 and L5B PCs in V2 (Fig. 3L–U, L2/3, *n* = 8 cells from 3 mice; L5B, *n* = 8 cells from 3 mice.). The CRACM of L2/3 and L5B PCs demonstrated V1→V2 projections differed from vM1-vS1 in that the median E/I ratio in L2/3 PCs was similar to that in L5B PCs (Fig. 3N,S, 0.19 vs 0.15, *P* = 0.11). This result suggests that long-range projections are not always generate a biased excitation in L2/3 PCs. In L2/3 PCs, the IPSC charges were significantly higher than EPSC charges at both soma (Fig. 3O, 0.4 ± 0.1 and 3.2 ± 0.6 for EPSC and IPSC, *P* < 0.01) and non-soma regions (Fig. 3O, 1.4 ± 0.1 and 8.8 ± 0.8 for EPSC and IPSC, *P* < 0.001). The E/I ratios were similar at both soma and non-soma regions (Fig. 3P, 0.11 vs 0.14, *P* = 0.23). The median neuronal E/I ratio was 0.13. In L5B PCs, the IPSC charges overweighed EPSC charges at both soma (Fig. 3T, 2.0 ± 0.4 and 11.8 ± 1.0 for EPSC and IPSC, *P* < 0.01) and non-soma regions (Fig. 3U, 7.6 ± 0.9 and 50.0 ± 4.9 for EPSC and IPSC, *P* < 0.01). The neuronal E/I ratios in V1→V2 were significantly lower than vM1→vS1 projections (0.16 vs 0.33, *P* < 0.01), suggesting that there is a regional difference of E/I ratios associated with long-range axonal activation, and perhaps related to primary vs. secondary sensory cortices. Application of TTX and 4AP abolished mixed synaptic inputs as well as di-synaptic involvement of interneurons (Fig. 3W–Y), with the presence of only perisomatic glutamatergic inputs (Fig. 3X).These results suggest that although there are some common features (e.g. inhibitory synapses are stronger in L2/3 PCs) shared by long-range projections across two sensory modalities, E/I ratios are largely different between vM1→vS1 and V1→V2. Thus confirming region and circuit specific nature of E/I ratios.

### Long-range inputs to PCs in paleocortices

**Figure 4:**
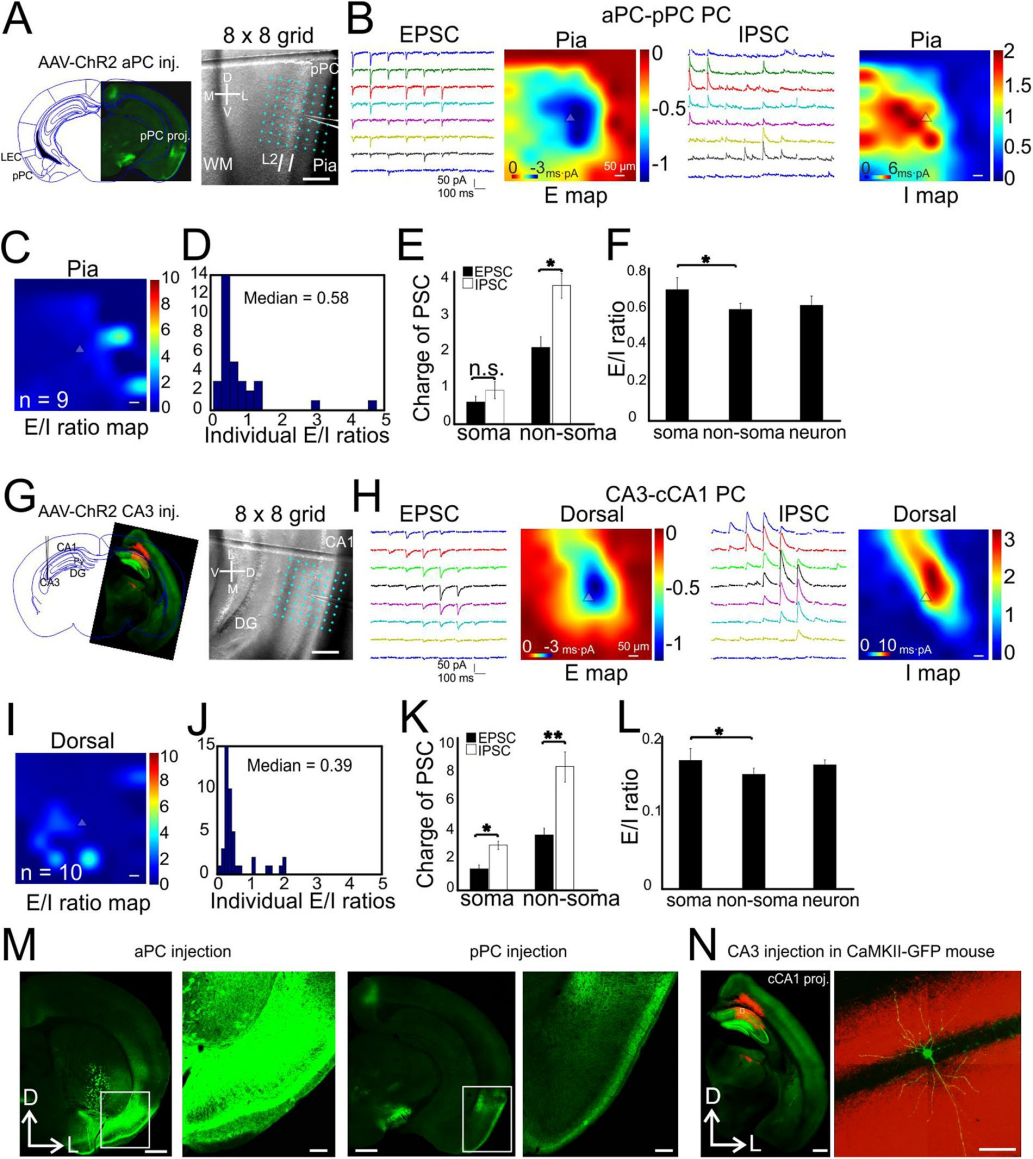
CRACM of long-range synapses on PCs in piriform cortex and CA1. (**A**–**F**) aPC synapses on L2 PCs in pPC. (**A**) Left, overlay of a slice with AAV-ChR2-expressing projection axons in pPC onto an atlas schematic (Bregma: −2.54 mm, Paxinos and Franklin, 1997). Right, A 8 × 8 photostimulation grid (75 µm spacing) overlaid on a coronal slice with an example of recorded PC in L2. (**B**) Traces: examples of EPSC/IPSC recorded from a single L2 PC. Heat maps: averaged CRACM input maps aligned by soma position. (**C**) Group averages of the E/I ratio maps across pixels. *n* = 9 cells, 2 mice. (**D**) Quantitative distribution of pixel E/I ratios shown in panel C. (**E**) Comparisons of absolute charges between EPSCs and IPSCs for both soma and dendrites regions. (**F**) E/I ratios at compartmental soma, dendrites regions and of the whole neurons. (**G**–**L**) CA3 synapses on contralateral CA1 PCs (stratum pyramidale). Legend layout similar as above. *n* = 10 cells, 3 mice. *P < 0.05, **P < 0.01, n.s., not significant. (**M**) Low and high magnification of one example of a coronal slice showing aPC injection and pPC projection. Scale bars in low-mag: 0.5 mm, scale bars in high-mag: 200 µm. (**N**) Virus injection in CA3 with GAD67-GFP mouse. Left, low-magnification image of ChR2-mChe expressing in contralateral CA1; right, Alexa-488 filled PCs during CRACM experiments. Scale bars: left 0.5 mm, right 50 µm.

Paleocortices, including both piriform cortex and hippocampus, have three layered structure and entirely different synaptic organization principals than the neocortex. We thus further examined the synaptic E/I balance to PCs in the paleocortices. AAV-CAG-ChR2 was injected in anterior PC (aPC) and we studied the synaptic E/I balance associated with the optogenetic activation of the long-range projections (>2 mm) from aPC to L2 PCs in posterior PC (pPC, Fig. 4A,M). The median neuronal E/I ratio was 0.6 (Fig. 4C,D, *n* = 9 cells, 2 mice), similar to vM1-vS1 L2/3 PCs (0.5). The charges of EPSC and IPSC were similar at the soma regions (Fig. 4E, 0.6 ± 0.1 and 0.9 ± 0.3 for EPSC and IPSC, *P* = 0.09), but not at the non-soma regions (Fig. 4E, 2.2 ± 0.4 and 3.7 ± 0.4 for EPSC and IPSC, *P* < 0.05). The E/I ratios were higher at the soma location than the non-soma regions (Fig. 4F, 0.7 ± 0.1 vs 0.6 ± 0.0, *P* < 0.05), with a neuronal E/I ratio of 0.6. We next expressed AAV-CAG-ChR2 in hippocampus CA3 region to study callosal projection from CA3 to contralateral CA1 (cCA1) PCs (Fig. 4G–L,N). The results demonstrated heavy basal dendritic targeting excitatory and inhibitory inputs (Fig. 4H). The median neuronal E/I ratio was 0.39 (Fig. 4I,J, *n* = 10 cells, 3 mice), similar to vS1→L5B PCs (Fig. 2I). The IPSC charges were significantly higher than EPSC charges at both soma (Fig. 4K, 1.3 ± 0.3 and 2.4 ± 0.6 for EPSC and IPSC, respectively, *P* < 0.05) and non-soma regions (Fig. 4K, 3.3 ± 0.5 and 8.2 ± 1.3 for EPSC and IPSC, respectively, *P* < 0.01). The E/I ratio at soma was significantly higher than non-soma regions (Fig. 4L, 0.6 ± 0.1 vs 0.4 ± 0.0, *P* < 0.05), with a neuronal E/I ratio of 0.4.

**Figure 5:**
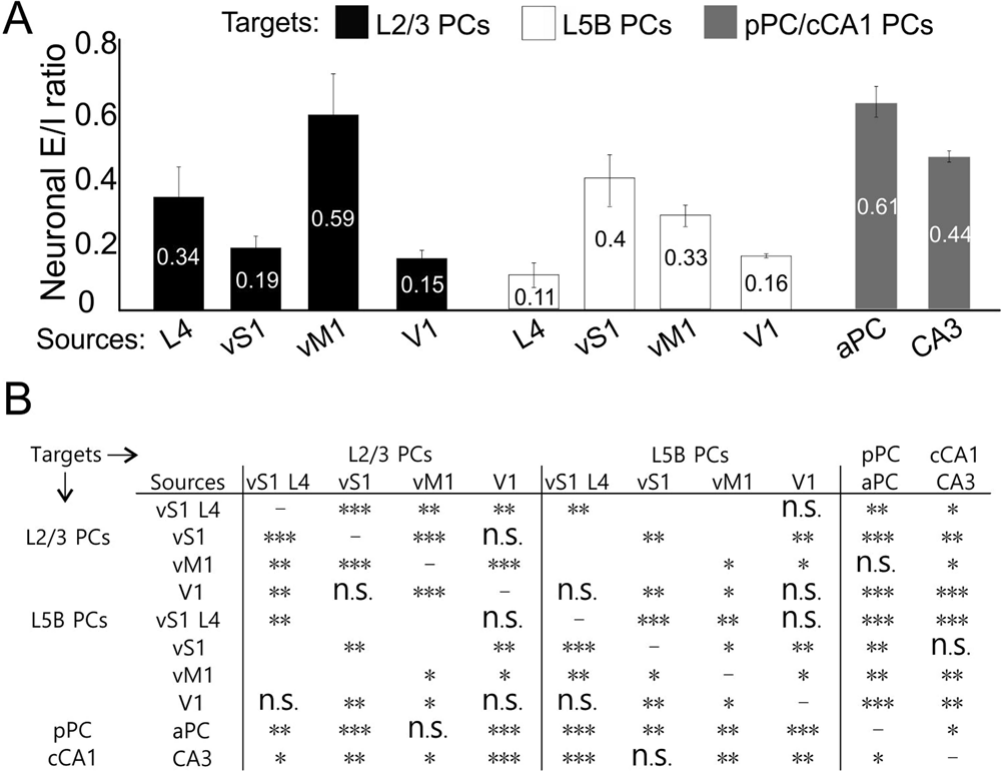
Summary of E/I ratios and comparisons across different recording targets and input cortical sources. (**A**) Neuronal E/I ratios across layers and cortical regions. (**B**) Statistical comparisons of neuronal E/I ratios between layers for same input sources and between same targets for different input sources. Nonparametric Wilcoxon signed-rank test, comparisons within same layers: L2/3: L4 vs vS1 *P* = 0.007, L4 vs vM1 *P* = 0.003, L4 vs V1 *P* = 0.006, vS1 vs vM1 *P* = 0.000, vS1 vs V1 *P* = 0.4782, vM1 vs V1 *P* = 0.000; L5B: L4 vs vS1 *P* = 0.000, L4 vs vM1 *P* = 0.003, L4 vs V1 *P* = 0.393, vS1 vs vM1 *P* = 0.047, vS1 vs V1, *P* = 0.008, vM1 vs V1 *P* = 0.011; comparisons between L2/3 and L5B: L4 *P* = 0.005, vS1 *P* = 0.004, vM1 *P* = 0.031, V1 *P* = 0.835; comparisons between neocortex L2/3 and paleocortex: L4 vs aPC *P* = 0.006, vS1 vs aPC *P* = 0.000, vM1 vs aPC *P* = 0.912, V1 vs aPC *P* = 0.000, L4 vs CA3 *P* = 0.043, vS1 vs CA3 *P* = 0.002, vM1 vs CA3 *P* = 0.039, V1 vs CA3 *P* = 0.000; comparisons between neocortex L5B and paleocortex: L4 vs aPC *P* = 0.000, vS1 vs aPC *P* = 0.009, vM1 vs aPC *P* = 0.002, V1 vs aPC *P* = 0.000, L4 vs CA3 *P* = 0.000, vS1 vs CA3 *P* = 0.751, vM1 vs CA3 *P* = 0.009, V1 vs CA3 *P* = 0.002; comparisons between aPC and CA3: *P* = 0.034.

The comparisons of neuronal E/I ratios from different layers or cortical regions were summarized (Fig. 5, see Legend for statistics). The results revealed several interesting aspects of cortical synaptic connectivity related to E-I balance. 1) Activation of long-range glutamatergic synapses typically generate higher E/I ratios in postsynaptic PCs (except V1-V2), especially for superficial layers in both neocortex (e.g. vM1→L2/3 0.6 vs vM1→L5B 0.3, *P* < 0.05) and paleocortex. 2) Activation of local recurrent glutamatergic synapses tend to excite L5B PCs whereas silence L2/3 PCs (e.g. vS1→L5B E/I ratio 0.4 vs vS1→L2/3 0.2, *P* < 0.01); L4 projections display the opposite properties: excite L2/3PCs whereas silence L5B PCs. 3) In several, but not all, pair-wise comparisons (inputs vs. postsynaptic), there were significant differences between somatic vs. non-somatic E/I ratios. 4) Despite the specificity and complexity of E-I balances in different regions and layers, there is universal subcellular wide overall E-I balance across all recording conditions (i.e. input sources, regions and layers, subcellular locations). This can be seen from all averaged E/I ratio maps in each recording condition. Most of the E/I ratio map has uniform color near median E/I ratio with a few noisy spots. These results suggest the relationship of synaptic excitation and inhibition depends on presynaptic sources and laminar positions of postsynaptic PCs as well as cortical regions in the brain. Further, the E/I ratio values vary at different subcellular compartmental locations (i.e. soma vs. dendritic regions, with exception of V1→V2 and vS1→vS1 L2/3), presumably resulting from distinct circuit wiring and correlated with unique computational rules of each circuit. For example, the strong di-synaptic recruitment of lateral inhibition in sensory pathways of vS1 may underlies sensory tuning.

### Contribution of SOM/PV INs to vM1→vS1 synaptic E/I balance

**Figure 6:**
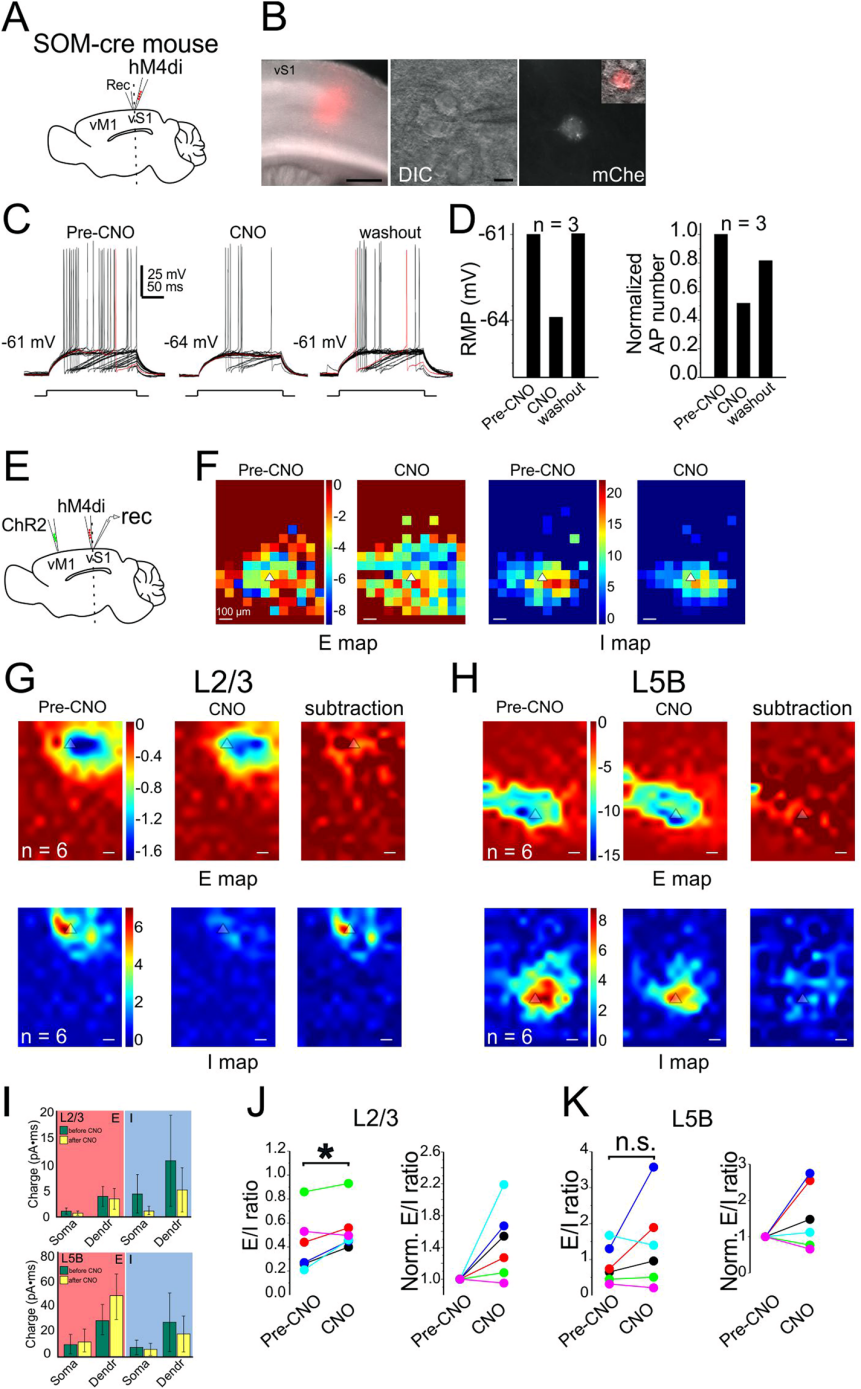
Contribution of SOM INs to vM1-vS1 synaptic E/I balance. Recorded L5B PCs in vS1 in hM4D DREADD-receptors experiments with SOM-cre mice. A coronal 300-µm-thick slice was cut and neurons were recorded in vS1 region. (**A**) Schematic showing injection and recording strategy. (**B**) left: fluorescent image of hM4D-receptors expressing neurons and their processes; middle: a DIC image showing a recorded PC in vS1; right: fluorescent image showing the recorded PC was SOM IN, inset showing a merged image. (**C**) The firing pattern of the recorded neuron in the panel B, before and during bath application of CNO. (**D**) Membrane potential and AP number changes for three SOM INs. (**E**) Schamatic showing ChR2 injection in vM1 and hM4D injection in vS1. Recording was performed in vS1. (**F**) A CRACM example of individual recording from a L5B PC in vS1 before and after CNO application. Note the E and I maps not changed obviously before and after CNO. Color bar before CNO also applying to maps after CNO. (**G**-**H**) CRACM of group data of L2/3 PCs (**G**) and L5B PCs (H), *n* = 6, 6 mice for both L2/3 and L5B. Color bars for Pre-CNO apply to CNO and subtraction maps. (**I**) Top, L2/3 PCs, charge comparisons of soma or dendrites (non-soma) before and after CNO application. Left, E comparison; right, I comparison. Bottom, L5B PCs, charge comparisons of soma or dendrites before and after CNO application. Left, E comparison; right, I comparison. (**J**) Comparison of the neuronal E/I ratio for L2/3 PCs before and after CNO application. (**K**) Comparison of the neuronal E/I ratio for L5B PCs before and after CNO application.

**Figure 7:**
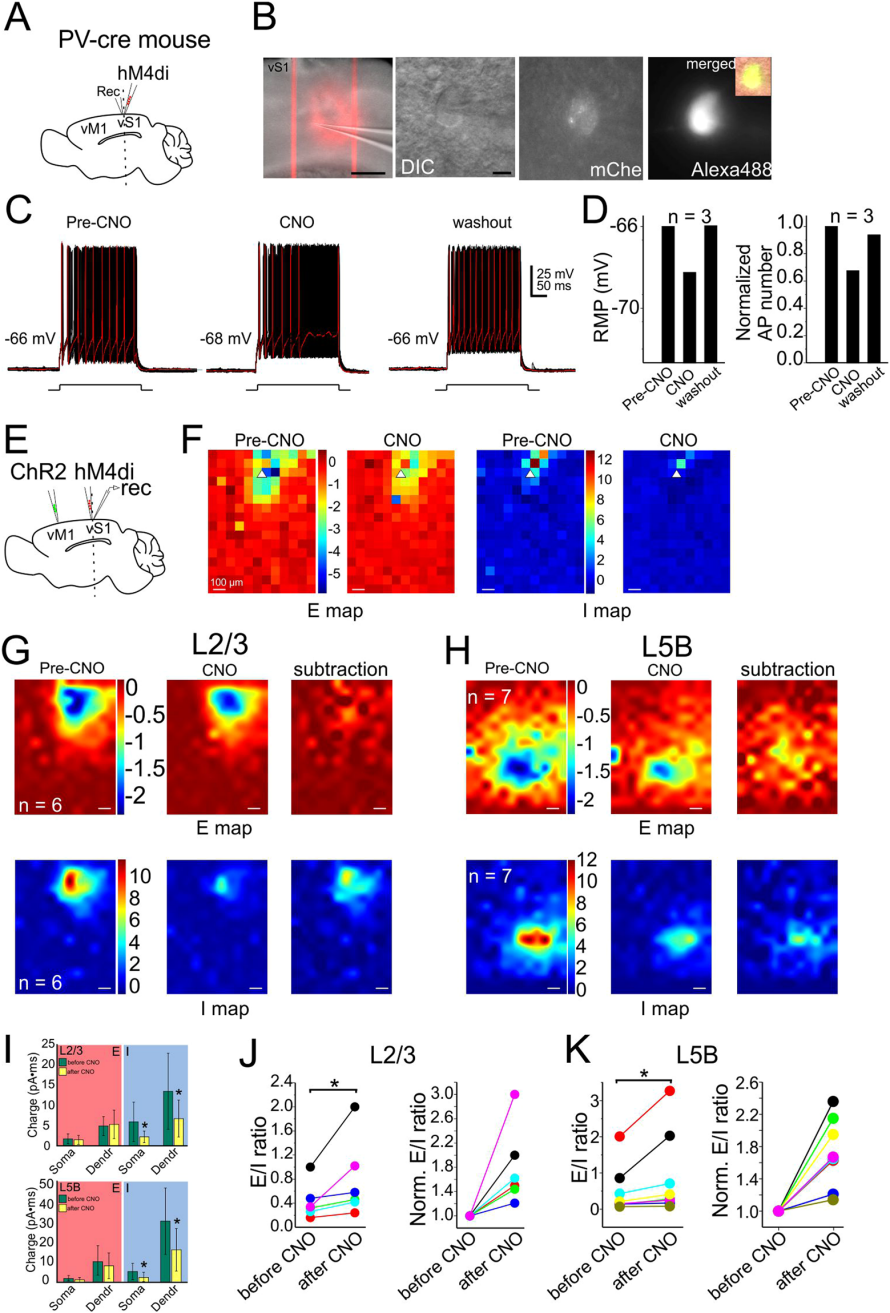
Contribution of PV INs to vM1-vS1 synaptic E/I balance. Recorded L2/3 PCs in vS1 in hM4D DREADD-receptors experiments with PV-cre mice. Legend layout similar to that of Fig. 6.

We hypothesized that the circuit and cell-type specific E-I balance was the result of unique contribution from different subtypes of INs. Due to the limited scope of the present study, we have focused on the role of SOM and PV INs in long-range vM1→vS1 mediated E-I balance. We examined this question by combining CRACM and chemogenetics (designer receptors exclusively activated by designer drugs, DREADD) in long-range vM1→vS1 synapses. To verify the silencing effect of hM4D, we expressed hM4D receptors specifically in SOM or PV INs in vS1 (Figs 6 and 7A,B) and recorded directly from these mCherry-positive INs (Figs 6 and 7C,D). Action potentials were recorded before and after clozapine N-oxide (CNO) application (100 µM)^36^, as well as 45 minutes after washout. Both the resting membrane potentials and the numbers of action potential decreased after CNO application and came back after washout (Figs 6 and 7C,D). In addition, CNO appeared to affect the frequency and amplitude of spontaneous IPSCs but not EPSCs of surrounding PCs (data not shown).

Next, we expressed ChR2 in vM1 and hM4D in vS1 SOM INs (Fig. 6E). CRACM was performed in vS1 PCs before and after CNO application in the hM4D DREADD expressing SOM-cre mice. For L2/3 PCs, the EPSC charge before and after CNO application did not show any difference (Fig. 6G,I top, *P* = 0.143 and 0.577, respectively). However, the neuronal E/I ratios increased significantly after CNO application (Fig. 6I,J, *P* = 0.042), suggesting that SOM INs were recruited by vM1→vS1 activation and may contribute to the maintenance of vM1→vS1 synaptic E/I ratio in L2/3 PCs. For L5B PCs, neither E or I charge showed significant change after CNO application (Fig. 6H,I,K). The comparison of neuronal E/I ratios before and after CNO application did not show any significant difference (Fig. 6K, *P* = 0.23). Thus, there is a layer specific modulation of vM1→vS1 synapses by SOM INs, and SOM INs appear to be important for long-range vM1→vS1 induced E-I balance in L2/3 PCs.

A similar set of CRACM experiments were then performed in PV-cre mice infected with hM4D DREADD. For long-range vM1→vS1 mediated E-I balance in L2/3 and L5 PCs, the comparison of E charge before and after CNO application did not display any significant difference (Fig. 7G). In contrast, the I charge of the soma or dendrites after CNO application decreased significantly (Fig. 7G,I and J L2/3: soma, *P* = 0.019 and dendrites, *P* = 0.049; L5B: soma, *P* = 0.001 and dendrites, *P* = 0.002). The neuronal E/I ratios of both L2/3 and L5B PCs increased significantly after CNO application (Fig. 7J,K, L2/3, *P* = 0.012; L5B: *P* = 0.023). In summary, PV INs were involved in vM1→vS1 synaptic E/I ratios for PCs in both layers (L2/3 and 5B) but SOM INs mainly contributed in L2/3. These results suggest that PV and SOM INs contribute differentially to long-range vM1→vS1 mediated E-I balances in different layers.

## Discussion

How should one interpret the EPSC and IPSC maps? It is first important to understand the limitations for this experiment: although ChR2 maybe expressed in specific regions, local blue light stimulation may have complex effects on ChR2 expressing neuronal components, such as dendrites, somata and axons. Thus the E and I map can not be simplified as one synaptic components. However, results from three separate experiments may help shed light into the understanding of these maps. (1) In vS1 injected slices, a small set of experiments were performed in ChR2-expressing PC cells in L2/3 (data not shown). In these cells, during the CRACM experiments, we first pointed the laser at the soma region of the recorded neuron and carefully reduced the laser intensity until it failed to elicit an action potential when laser beam was delivered to the soma region. We then completed the CRACM experiments and acquired EPSC and IPSC maps. These maps were similar to the maps of L2/3 non-ChR2 expressing PC cells (e.g. Fig. 2A) in the same slices. We speculate that at relatively low laser intensity, small ChR2 currents induced near the perisomatic area may not be sufficient to bring a PC to fire, however similar amount of currents near the synaptic terminals (or axons) may be sufficient to induce synaptic release. This is presumably because of the distinct biophysical properties in the synaptic terminals, i.e. smaller membrane capacitance (~100 folds smaller than soma), larger membrane resistance, and thus a transient light-induced ChR2 mediated inward current in these structures will generate much larger membrane depolarizations than the soma and dendritic areas. These larger depolarizations are perhaps sufficient for activation of voltage-gated calcium channels in the terminals. This notion was originally proposed in the original sCRACM experiments (in the presence of TTX)^33^: the low intensity laser induced ChR2 mediated inward current failed to induce synaptic release at the synaptic terminals, however, after the researchers added 4-AP (100 μM) which blocks A-type potassium channels, then the same laser induced ChR2 mediated current was sufficient to induced synaptic release from the nerve terminals. Petreanu *et al*., proposed that direct light activation of synaptic terminals, rather than propagation of depolarizations are responsible for the synaptic releases. (2) In the vS1 L4 experiments (i.e. Fig. 1), PCs in L4 were transfected with AAV-flex-ChR2 in Scnn1a-Tg3-cre mice while recordings were made in L2/3 cells. L4→L2/3 represents the canonical sensory feedforward circuits within S1. Our results show that in L2/3 PCs, both E and I maps were largely confined within L2/3 (Fig. 1A). This result suggests that although the L4→L2/3 projections originate from L4, there were no laser induced responses in L4. Therefore, these results also support the notion that at low laser intensities, ChR2 mediated local currents are sufficient to trigger the synaptic release from synaptic terminals but may not be sufficient to trigger action potential in *en passant* axons or in the soma and dendrites. (3) In our chemogenetic experiments (Figs 6 and 7), bath application of clozapine N-oxide (CNO,100 µM)^36^, reduced action potential numbers (e.g. Figs 6C and 7C). However, the effects of CNO were interesting: it had very modest effects on action potential inductions and predominantly reduced near threshold current induced APs (e.g. Figs 6C and 7C). In SOM-cre mice infected with hM4D DREADD, we found that bath application of CNO nearly wiped out the entire IPSC maps in S1 L2/3 PC induced by CRACM activation of the long-range M1→S1 glutamatergic inputs but had little effects in L5B PCs(Fig. 6G vs. H). This differential effects of CNO on I map of L2/3 vs. L5B PCs support the idea that inhibitory maps are results of delicate long-range glutamatergic depolarization of SOM INs in L2/3 PCs Thus we interpret our E and I maps as primarily representing the synaptic terminal field of specific pathways transfected with ChR2. For di-synaptic inhibitory maps, they represent the glutamatergic terminal field and their innervation of GABAergic interneurons. For example, in Fig. 1B, inhibitory maps in adjacent barrels of L23 represent the locations (and strength of their synaptic innervation of target neurons) of interneurons recruited by L4-L2/3 glutamatergic inputs. However, we cannot totally rule out the possibility that either direct activation of somas (e.g. interneurons) or en passant axons by ChR2 contribute to the E and I maps, although this possibility is relatively low.

Previous studies of synaptic connections between cortical cells within or across layers or regions largely focuses on comparing strength of monosynaptic transmission^33,35,37^. While monosynaptic connections within a brain provide essential knowledge of circuit organization, normal brain acts as a result of intensive interactions of numerous cortical elements through the coordinate efforts of mono-, di-synaptic and poly-synaptic communications^2–4^. How excitatory and inhibitory synapses converge on pyramidal cells (PCs) across different regions determines the logic of neural computation. We thus investigated neuronal/compartmental E-I balance of PCs in primary (S1) and secondary (V2) sensory cortex and paleocortices (piriform cortex and hippocampus CA1) induced by defined presynaptic sources in adult mouse brain slices.

Our results show that within S1, inputs from different sources (e.g. local vs. long-range) on the same neurons, or the same inputs (e.g. vM1→vS1 input) formed on different targets (i.e. L2/3 vs. L5) induced different E/I ratios in the postsynaptic PCs. Evoked E/I ratios in different regions (e.g. paleocortices vs. neocortices) were largely different. The phenomenon of E-I balance has been well documented in many brain regions. For example, in V1 of cat, E-I conductances evoked by visual stimuli are balanced in neurons within the orientation map^21^. In the mouse V1, L2/3 pyramidal cells show stable E/I ratios in time despite fluctuating cortical activity levels^16^. In the prefrontal cortex, E-I balance is maintained during the “up” states^10^. In primary auditory cortex of rat, E-I balance exhibit a strong linear correlation with the inputs^11^. The majority of these studies focused on evoked E-I conductances in single neurons from single cortical regions in response to a single source of input. To our knowledge, vertical studies that examining E-I balances across multiple layers, or the same layers between different inputs and horizontal studies examining E-I balance between different brain regions are largely unavailable. A recent study^38^ by Adesnik and Scanziani made vertical comparisons of E-I balance in vS1 and V1 across layers and found that horizontal projections suppress superficial layers while simultaneously activating deeper cortical output layers^38^. This layer-specific modulation is in agreement with our results regarding layer specific E-I ratio in vS1. In addition, our data expanded the conclusions regarding E-I balance by showing that the layer–specific E/I ratio is also input-specific and brain region-specific.

Within vS1, the finding of stronger L4→L2/3 PCs and weak L4→L5B PCs is also consistent with previous findings using pair recording, glutamate uncaging or CRACM^33,39–42^, supporting a stronger feedforward L4→L2/3 projection. Furthermore, lateral inhibition from adjacent columns was stronger in L5 PCs than in L2/3. This results support the observation that adjacent whisker deflection suppresses the principal whisker *in vivo*^43^. Interestingly, the asymmetric E and I maps in 2D space were clearly observed for L4-specific input among all tested circuits. Intracortical neuronal interactions clearly add more complexity and constraints to local circuit computation on top of L4-specific input. The asymmetry was also reflected in that long-range synapses displayed larger E map size than its counterpart (I map), suggesting a potentially active role in local neural computation. The low E/I ratios found in vS1-L2/3 PCs is consistent with previous data in that L2/3 circuit is characterized with a recurrent excitation, typically matched by a stronger di-synaptic inhibition^4,31,44,45^. In contrast, higher E/I ratios in L5B PCs may result from an amplification of strong feedforward excitatory input from L2/3 PCs and L5 INs^46,47^. Alternatively, it may possibly result from an intralaminar recurrent circuit between L5B PCs^48^.

Besides local synaptic microcircuits, distal reciprocal connections between vS1 and other cortical regions including vM1, S2 and thalamus, underlies important functions such as sensorimotor integration and associative learning^49^. Activities in vM1 typically lag 8 ms behind vS1 activities^50^. Long-range projections from vM1 to vS1 mainly target on L2/3 and L5/6^32^. Higher E/I ratio found in vM1-vS1 L2/3 PCs thus indicates strong influence of vM1 on vS1 supragranular layers. Interestingly, a study mapping vS1-vM1 synapses found vS1 preferentially innervates on superficial layers of vM1^51^, suggesting a circuit basis favoring superficial layer interaction underlying somatosensory integration. The E/I ratio in vM1-vS1 L5B PCs was lower compared to vM1-vS1 L2/3 PCs but comparable to E/I ratios in vS1-L5B PCs and L4-L2/3 PCs. Long-distance synapses from aPC-pPC and from CA3-cCA1 both displayed high E/I ratios in PCs, suggesting that higher E/I ratios may be characteristic for many long-range projections. An exception to this rule is the V1-V2 synapses (which had lower E/I ratio), suggesting a different computation in the visual system. One possible explanation could be the difference in the density of horizontal axon collaterals, which is relevant to amplification of polysynaptic and postsynaptic depolarization^52^.

E-I balances are induced by balanced di-synaptic and poly-synaptic activation of GABAergic and glutamatergic cells. Despite a wealth of knowledge regarding how PV- and SOM-INs differentially innervates dendritic and perisomatic area of PCs^53^, limited data is available regarding the contribution of PV and/or SOM-INs to mixed synaptic conductances. In the mouse V1, L2/3 PCs show stable E/I ratios in time despite fluctuating cortical activity levels. The E-I balance is mediated exclusively by PV, but not SOM-INs^16^. Similar function of PV neurons in regulation of receptive field of L2/3 PCs has been reported in auditory cortex^54,55^, instead, SOM-INs are involved in delayed regulation of receptive field properties. Thus, although the data is limited, previous studies reveal differential contribution of PV and SOM-INs to E-I balances. Applying chemogenetic to silence specific INs while optogenetically activating long-range vM1 inputs to vS1, we demonstrate a differential contribution of PV and SOM-INs to the E-I balances in a postsynaptic target-specific manner (L2/3 vs L5B): as expected, PV-INs predominantly contribute to the somatic inhibition in both L2/3 and 5B; and unexpectedly, SOM-INs predominantly affecting E-I rations of L2/3 but not L5B PCs.

Despite the specificity and complexity of E-I balance in different regions and layers, there is an overallsubcellular wide E-I balance across all inputs, cell types and regions. Our results thus demonstrate that there are both universal cell-wide E-I balance and high specificity in the E/I ratios within each circuit^56^. The relative spatial size of E and I map roughly predicts the cell-wide E/I ratios (e.g. compare Fig. 2B vs G). It has also been shown that the number of excitatory and inhibitory synapses across individual dendrites maintains a constant ratio *in vitro*^57^. Together, these results suggest that there are coordinated excitatory and inhibitory synaptic wiring across various cortical layers/columns and modalities, which presumably arise during development and may underlies circuit-specific neural computations. This is an interesting and remarkable results, and it suggests a highly organized efforts during development to establish the balanced synaptic in all different subcellular locations, and during adult stage to maintain this very well balanced connections. The detailed mechanisms by which E and I form a uniform balance remain elusive. However, this highly organized level of specificity and generalization will form the basis for further understanding of disease-specific pathological circuit changes.

## Materials and Methods

### Animals

All experiments were performed under protocols approved by the Institutional Animal Care and Use Committee (IACUC) and the Biosafety Committee of the University of Wyoming. All experiments were performed in accordance with guidelines and regulations from the National Research Council of the Academy of Sciences and NIH GUIDELINES FOR RESEARCH INVOLVING RECOMBINANT OR SYNTHETIC NUCLEIC ACID MOLECULES. Mice were housed in a vivarium maintained at 22–23 °C on a 12:12 h light-dark cycle. Food and water were available *ad libitum*. Scnn1a-cre, CamKII-GFP and CD1 mice were obtained from Jackson Laboratories (Bar Harbor, ME). Both male and female mice aged postnatal day 45–150 were used in this study.

### Stereotaxic viral injection

Virus injection was performed in mice aged P14-P16. Mice were anesthetized with 2% isoflurane (vol/vol) and maintained with oxygenated 1% isoflurane throughout surgery procedure. AAV2.1.CAG.hChR2(H134R)-mCherry (University of Pennsylvania Vector Core) was used for GAD67, CamKII-GFP or CD1 mice for non-specific neuronal transfection. AAV2/1.CAGGS.flex.ChR2.tdTomato.WPRE.SV40 (University of Pennsylvania Vector Core) was used for Scnn1a-cre mice for L4 excitatory neurons-specific transfection, which allows us to examine E-I balance associated with optogenetic activation of L4→L2/3 and L4→L5B connections inputs in vS1. Viral vector was loaded into the tip (~20 µm in diameter) of a beveled glass micropipette (Drummond Scientific Co.). A custom stereotactic apparatus was used to deliver viral vector to cortex through a small hole drilled into the skull. The coordinates were all relative to Bregma in mm. For vS1 injection (posterior 1.0, lateral 3.0), vM1 injection (anterior 1.0, lateral 0.9) and V1 injection (posterior 3.6, lateral 2.5), virus was injected at two depths: 400 µm and 800 µm; for vS1 L4 injection, 400 µm and 600 µm. The virus typically spread to ~1 mm away from center and covered 4–6 barrels (e.g.Figs 1–2A). The coordinate for aPC injection was anterior 1.7, lateral 2.7 and ventral 3.6. The coordinate for CA3 injection was posterior 2.4, lateral 2.7 and ventral 2.4. For each depth, a volume of ~150 nl was injected within 1–2 minutes using a micromanipulator (MP-285-system, Sutter Instrument). Injection pipette was kept in place for 5 minutes for each depth after injection. Injected mice were put back to dam after recovery from anesthesia in a separate cage with a heating pad underneath. After weaning day, pups were separated by gender until experiments. Electrophysiological experiments would not start until at least 4 weeks after viral injection.

### Slice preparation and electrophysiology

Mice aged P46–180 was decapitated under isoflurane anesthesia and the brain quickly put in ice cold cutting solution (in mM: 2.5 KCl, 1.25 NaH2PO4, 10.0 MgCl2, 0.5 CaCl2, 26.0 NaHCO3, 11.0 glucose and 234.0 sucrose). Coronal brain slices were cut (TPI, St. Louis, MO) in 300-um thickness, incubated in 35 °C oxygenated (95% O2 and 5% CO2) aCSF (in mM: 126.0 NaCl, 2.5 KCl, 1.25 NaH2PO4, 1.0 MgCl2, 2.0 CaCl2, 26.0 NaHCO3 and 10.0 glucose, 295 mOsm) for 50 to 60 minutes. In a subset of experiments, thalamocortical slices were used and E/I ratio did not differ significantly compared to coronal slicing. Incubation chamber was then put in room temperature for up to 5 h throughout experiments. Recordings were performed at room temperature and a selected slice with prominent barrels was immersed in circulating (1 ml/min) bubbled aCSF. Slices were always positioned in a similar orientation in the recording chamber. Borosilicate glass patch pipettes with filament were pulled with a P-97 puller (Sutter Instrument) generating a resistance of 4–5 MΩ. Recording pipettes were filled with a Cs-based biocytin-included (0.5%, wt/vol) intracellular solution (in mM: 120 cesium gluconate, 10 phosphocreatine-Tris, 3 MgCl2, 0.07 CaCl2, 4 EGTA, 10 HEPES, 4 Na2-ATP, and 1 Na-GTP) with pH adjusted to 7.35 with CsOH (289 mOsm). Whole-cell recordings were made with an Axopatch 700B amplifier (Axon Instruments) and a 1322 A board. Data from CRACM experiments were collected using Matlab-based software Ephus^58^. Cells below pia surface ranged 50 to 110 µm were collected. For CRACM, EPSCs were recorded in voltage clamp with holding potential at −45 to −48 mV (L2/3 cells) or −45 to −55 mV (L5B cells) such that a short-duration (several ms) downward current was evoked when a laser beam was directed to recorded neuronal soma position. IPSCs were recorded in same mode with holding potential at 0 mV. Cells with series resistances ranged 15–40 MΩ and with less than 15% resistance change throughout experiments were included for subsequent analysis. Using IR/DIC visualization and epifluoresence microscopy, L2/3 PCs were targeted through recording of GFP-expressing cells in CamKII-GFP mice or, in CD1 mice by examining the morphology of typical principle cells and larger capacitance compared to INs, followed by post hoc examination of reconstruction of biotin-filled cells. In addition, PCs typically display high-frequency and -amplitude IPSCs compared to INs. L5B PCs (pyramidal tract neurons) were identified as above in addition to the existence of thick proximal dendrites typical for L5B PCs (e.g. Fig. 3W).

CRACM The blue laser beam (Shanghai Laser & Optics Century Co., 473 nm) was carefully aligned with routing mirrors, Pockels cell (ConOptics) and a pair of galvanometer scanners to generate a relatively cylindrical beam through a 4× objective (0.10 NA, Olympus) at the specimen plane (~15 µm in diameter, full-width at half max) as reported^28,30^. A portion of laser beam was redirected to a photodiode (silicon detector, Edmund Optics) through a beam-splitter (30:70 split, 450–650 nm range, Thorlabs). The detected voltage was coupled to laser intensity measurement using a handheld laser power meter (Edmund Optics) placed at the immediate back of the 4× objective. The majority of recorded neurons were measured under two different intensities. The laser power (0.1–1.6 mW, 1 ms duration) was adjusted such that the largest EPSC_CRACM_ had peak values ranged 30–50 pA. Cells with less than 10 pA peak IPSC_CRACM_ at maximum laser intensity were excluded from subsequent analysis (*n* = 4). Each cell was mapped with a 12 × 16 (for vS1 and V2 recording) or 8 × 8 (for piriform and CA1 recording) photostimulation grid (distance between adjacent points, 75 µm, Fig. 1A) capturing the majority, if not all, polysynaptic inputs to dendritic arbor of recorded neurons. Each map was repeated 3 times. The photo-stimulation sequence was given in a pseudo-random manner to maximize the intervals between adjacent photo-stimulation grid spots^33,34,51,58^.

### Data analysis

We performed data analysis using custom programs written in Matlab (MATLAB, MathWorks). Each trace of raw data in Ephus represented a 400-ms time window of averaged recorded current under voltage clamp mode: first 100 ms was the baseline before photostimulation (time 0), laser delivery at 100 ms and the following 300 ms showed the evoked EPSC/IPSC. We calculated the area of the postsynaptic potential using Matlab and defined the area as the integral of the recorded potential above baseline between 0 and 200 ms after laser onset. CRACM map pixel values corresponded to the average EPSC or IPSC charge calculated as above. This time window essentially captured the mixture of monosynaptic and major components of mixed activities of evoked EPSC/IPSC. We used the EPSC/IPSC charge within this time window to calculate their corresponding strength and their ratios. Using the peak amplitude as a measurement tended to generate a larger E/I ratio compared to charge (data not shown). For each pixel of a CRACM map (16 × 12 = 192 pixels, or 8 × 8 = 64 pixels), we calculate the charges for both EPSCs and IPSCs. The actual charges were provided (e.g. Fig. 1B,H, inset scale), and the E and I maps were also normalized to the maximum E charge within maps (e.g. Fig. 1B,H, color bars). E/I ratios were calculated by dividing the EPSC charge by IPSC charge (pixel E/I ratio, e.g. Fig. 1C). This analysis gave rise to a spatial distribution pattern of E/I ratios of one recorded neuron across its dendritic arbor through mixed synaptic input. The ratio limit was arbitrarily set as 10 (E/I ratio >10 forced to 10, e.g. Fig. 1C) to reveal compartmental distributions of spatial individual E/I ratios. We further defined a “soma” region as the rectangular six pixels with the recorded soma at the center (Fig. 1A, red pixels). Other pixels in blue with evoked responses were defined as “dendrites” region (Fig. 1A, blue pixels). Typically, laser-induced responses occurred in only a subset of blue pixels, reflecting the spatial “receptive field” of recorded cells. In addition, for each recorded neuron, one neuronal E/I ratio could be derived from dividing the sum of individual pixel EPSC charge by the sum of individual pixel IPSC charge within a map. This calculation generated an overall excitability profile of recorded cells, but discarding the spatial information of relative E/I strength at each pixel location. EPSC maps of each cell were normalized to the maximum pixel charge within the same cell and IPSC maps then normalized to the EPSC. CRACM maps were further averaged across cells within a class (e.g. Fig. 1B).

Nonparametric Wilcoxon signed-rank test was used for E/I ratio comparisons as well as PSC charge comparisons between paired groups. ANOVA was used for multiple group comparison.
